# Healthcare Professionals’ Ratings and Views of Person-Centred Care in the Context of Allogeneic Hematopoietic Stem Cell Transplantation

**DOI:** 10.1177/11786329241310735

**Published:** 2025-01-02

**Authors:** Anna O’Sullivan, Jeanette Winterling, Annika Malmborg Kisch, Karin Bergkvist, David Edvardsson, Yvonne Wengström, Carina Lundh Hagelin

**Affiliations:** 1 Department of Health Care Sciences, Marie Cederschiöld University, Stockholm, Sweden; 2 Department of Nursing, Sophiahemmet University, Stockholm, Sweden; 3 Department of Neurobiology, Care Sciences and Society, Division of Nursing, Karolinska Institutet, Stockholm, Sweden; 4 Karolinska Comprehensive Cancer Centre, Medical unit HHLH, Karolinska University Hospital, Stockholm, Sweden; 5 Department of Health Sciences, Lund University, Lund, Sweden; 6 Department of Hematology, Oncology and Radiation Physics, Skåne University Hospital, Lund, Sweden; 7 Department of Public Health and Caring Sciences, Uppsala University, Uppsala, Sweden; 8 School of Nursing and Midwifery, La Trobe University, Melbourne, Australia; 9 Sahlgrenska Academy, Institute of Health and Care Sciences, University of Gothenburg, Gothenburg, Sweden; 10 Breast Cancer Centre, Karolinska Comprehensive Cancer Centre, Karolinska University Hospital, Stockholm, Sweden

**Keywords:** Person-centred care, patient-centred care, allogeneic stem cell transplantation, healthcare professionals, healthcare research, health service delivery, health service evaluation

## Abstract

**Introduction:**

Allogeneic stem cell transplantation (allo-HCT) involves a long trajectory with high risk of complications. In person-centred care (PCC), patients’ needs, resources and the care relationship are central to the care process. Healthcare professionals’ (HCPs) ratings of PCC have not previously been investigated in this context.

**Objectives:**

The aim of this study was to investigate healthcare professionals’ ratings and views of person-centred care in allo-HCT care, and associations with individual characteristics and targeted PCC education.

**Design:**

Cross-sectional study, employing quantitative and qualitative methods.

**Methods:**

85 HCPs at two Swedish allo-HCT centres participated (80% women; mean age: 44 years, range: 23-72 years). A survey was conducted using the PCC Assessment Tool (P-CAT), containing 13 items, a total scale (min 13-max 65) and two subscales (I: min 8-max 40; II: min 5-max 25). Additionally, HCPs’ written responses to four study-specific questions about PCC were collected.

**Results:**

The mean for P-CAT total scale was 45.31, (subscale I: 28.41; subscale II: 16.90). Higher ratings of PCC were reported for assessment of patients’ needs, discussion about how to provide PCC and patients’ care, while time to provide PCC, the care environment and how the organization prevents providing PCC were rated lower. Higher age and targeted PCC education were associated with higher PCC ratings. HCPs described PCC as the patient being seen as a capable individual with their own resources, with PCC increasing patient and family involvement—giving higher satisfaction and tailored care for patients. However, HCPs reported time as a barrier for PCC.

**Conclusion:**

HCPs’ ratings of PCC in this context are high regarding discussing and assessing patients’ needs, but there is room for improvement regarding organizational and environmental aspects. Targeted PCC education increases the level of PCC. HCPs’ views of PCC partly reflect the foundations of PCC—patient’s narrative, capability and involvement.

## Introduction

An increase in the number of allogeneic hematopoietic stem cell transplantations (allo-HCT) has been seen over the last decade, with more than 80,000 procedures performed internationally each year—a figure that is estimated to reach 1 million by the end of 2024.^1^ Allo-HCT is an advanced medical treatment aiming to cure fatal diagnoses, commonly hematological malignancies, requiring an individually matched donor. Approximately 300 transplantations are performed yearly at six transplant centres in Sweden.^2^ The treatment trajectory includes a hospitalization of 4 to 6 weeks, with restrictions due to the intensive treatments and high risk of infections. Discharge is followed by a three-month intensive period of care and lifelong follow-up care thereafter. The outcome of the treatment is often uncertain and medical complications are common, for example, infections and the immune reaction Graft versus host disease (GvHD).^3^ Patients experience physical and psychological symptoms as well as social and existential distress during the entire trajectory.^4,5^ Post allo-HCT patients experience functional impairment as well as psychological distress related to an uncertain future with risk of relapse and complications.^6^

Person-centredness and person-centred care (PCC) are important emerging perspectives in health care that acknowledge the person in need of care.^7,8^ PCC is a philosophical approach to health care on which the healthcare system must be based and where the person is central.^9,10^ In PCC, the person is not just their illness; this type of care considers the person’s resources, circumstances, and obstacles. Respect for the person’s integrity and mutual security, understanding, and trust in the care relationship are key aspects of PCC. There is a broad range of how national study programs in Sweden have incorporated PCC in education for healthcare professions. Content referring to person-centred care has been found in about a sixth of local course syllabuses, with differences between and within programs.^11^ Implementation of PCC in Swedish higher education is ongoing, even though seemingly fragmented and driven by individuals and there is uncertainty around the meaning and value of PCC and how to implement it.^12^ There are free online educations about PCC available, for example, through the University of Gothenburg Centre for Person-centred Care—GPCC and through the Swedish nursing association.

The clinical advantages with PCC have been investigated previously in different contexts, for example, in severe coronary conditions PCC has been found to improve patients’ health-related quality of life.^13,14^ A study in nursing homes showed that a more person-centred climate is associated with higher ratings of the quality of care.^15^ Studies focusing on PCC in cancer care found that PCC improves patients’ care outcomes, preparedness for treatment, and safety, as well as the quality and safety of hospital care.^8,16,17^ A systematic review about patients perceived quality of person-centred care, mainly based on studies within hematological malignancies, showed that factors such as respect of patients’ values and preferences, emotional support, management of psychological needs, as well as integrated and coordinated care received a lower score. Furthermore, patients’ perceptions of the quality of PCC were impacted by clinical factors such as type of malignancy, psychiatric comorbidities, time interval of diagnosis and type of treatment.^18^

A review about PCC and HCPs’ job satisfaction and work-related health included mainly studies in the context of care of older people and a positive association between PCC and healthcare provider outcomes was seen, although the tools used to evaluate outcomes varied greatly.^19^ HCPs’ ratings of PCC in care of older people have shown, for example, that higher levels of staff satisfaction, lower levels of job strain and lower levels of work-related stress were all associated with higher ratings of PCC.^20^ Another study showed that job satisfaction and well-being were associated with higher ratings of PCC in community care nurses; however, the association between job strain and higher ratings of PCC was observed to be a negative one.^21^ Previous studies in other contexts have indicated the benefits and importance of PCC in care. However, studies about HCPs’ experiences of PCC in the context of allo-HCT are sparse and, therefore, the aim of this study was to investigate healthcare professionals’ ratings and views of person-centred care in allo-HCT care, as well as associations with individual characteristics and targeted PCC education.

## Methods

### Design

A cross-sectional study was conducted, with a survey including HCPs from two allo-HCT centres in Sweden to investigate HCPs’ ratings of PCC. To deepen the understanding of HCPs’ ratings of PCC and to investigate their views of PCC further was qualitative data in the form of written answers to open-ended questions about PCC collected from HCPs at one of the allo-HCT centres.

### Setting and sample

There are six centres in Sweden that perform allo-HCTs, the two largest centres are involved in a research project in which we intend to implement and evaluate the effects of a model for PCC. The two largest centres partook in this study, one of the centres carries out allo-HCT as well as other treatments in an adult haematological clinic, and around 60 allo-HCTs are performed per year. The other centre carries out allo-HCT on both paediatric and adult patients in the same ward, and they only focus on allo-HCT and cell therapy, around 100 allo-HCTs per year are performed there. Both centres are staffed by registered nurses, assistant nurses, and physicians on a permanent basis, with access to physiotherapists, dieticians and counsellors when needed.

The inclusion criteria for this study were: HCPs who were working at one of the two centres and had patient contact. HCPs who had responded that they had no patient contact were excluded from the study. The two centres had partaken in a targeted PCC education developed within the research project. The targeted PCC education included material about PCC, some produced within the research project of the current study as recorded power-point lectures about essential parts of PCC based on the literature, and some publicly available material about PCC. The targeted PCC education also contained group discussions about PCC in general and related to the clinic where the HCPs work. The timing of the targeted education and the course of action for giving the education to HCPs differed slightly between the two allo-HCT centres. At both centres nurses and mangers involved in the project took part of the targeted PCC education. Additionally, the targeted PCC education was given to HCPs at the centres at different educational days over a time period at the end of 2022 and the beginning of 2023. This study consists of two samples, one sample is HCPs at both centres who were invited to take part in the survey. The other sample consisted of HCPs at one of the centres, who took part in the targeted PCC education during an annual education day and gave their written responses to questions about PCC. The education in PCC is only included as a contextual description and had no bearing on the design of the study.

### Data collection

An invitation to participate in the study was sent via email to a total sample including all eligible HCPs at the two centres (*n* = 231) in December 2022 by administrative staff at each site, followed by two reminders at the beginning of 2023. The invitation contained a link to the survey; information about the study, including researchers’ contact information; and information stating that the study was performed in cooperation with the hospital in which they worked. The written information assured confidentiality and the right to withdraw from the study at any time without explanation. A filled-out online survey was considered as consent to participate. The data collection with the survey took place over three months to maximize participation. No power calculation for the sample size was done for this study, since all eligible participants were invited to take part in the survey.

Additionally, HCPs’ (*n* = 41 HCPs) written responses (*n* = 170 responses, 1-4 responses/participant) to four study-specific open-ended questions about PCC (Table 1) were collected on three different occasions during a single annual education day, which contained the targeted PCC education, among other subjects, at one of the participating centres during the autumn of 2022.

**Table 1.** Description of all variables and subscales for P-CAT, as well as the open-ended questions.

Items and subscales	Responses	Data collection
Subscale 1 – Extent of personalised care: 1. We often discuss how to give PCC 2. We have formal team meetings to discuss patients’ care 3. The life history of the patients is formally used in the care plans we use 4. The quality of the interaction between HCPs and patients is more important than getting the tasks done 5. We are free to alter work routines based on patients’ preferences 6. Patients are offered the opportunity to be involved in individualized everyday activities 11. Assessment of patients’ needs is undertaken on a daily basis 13. Patients are able to access outside space as they wish	Agree completely - Disagree completely	Survey
Subscale 2 – Amount of organisational and environmental support: 7. I simply do not have the time to provide PCC 8. The environment feels chaotic 9. We have to get the work done before we can worry about a homelike environment 10. This organisation prevents me from providing PCC 12. It is hard for patients in this facility to find their way around	Agree completely - Disagree completely	Survey
What is PCC according to you?	Open-ended	Annual education day
What benefits do you see with PCC?	Open-ended	Annual education day
What is your view on how you work with PCC at your clinic today?	Open-ended	Annual education day
What are the barriers to work in accordance with PCC at your clinic today?	Open-ended	Annual education day

### Measures

The PCC Assessment Tool (P-CAT) is a self-reporting assessment scale for staff ratings of person-centredness in their practice. It was first developed by Edvardsson et al.^22^ and has since been validated and used in other studies and in different settings.^23,24^ The instrument contains 13 items on a 5-point Likert-type scale (ranging from 1 = “*Disagree completely*” to 5 = “Agree completely”) (Table 1). The total scale ranges from 13 to 65, where higher values indicate a higher degree of person-centeredness. No cut-off values for high and low person-centeredness were described in the original publication. The tool has two subscales: Subscale I—Extent of personalized care (range: 8-40) and Subscale II—Amount of organizational and environmental support (range: 5-25). Subscale I contains statements about individualized care performed by health care professionals and subscale II is made up of statements focusing on organizational and environmental arrangements in the care setting. For the data collection the Swedish version was used, validated by Sjögren et al.^23^ Background variables used in the study were the participants’ age, sex, education, profession, years in the profession, workplace and whether the participant had taken part in any education about PCC provided through the current research project. The survey also consisted of other questions, not included in the current study, focusing on organizational readiness including work environment and job satisfaction. The survey consisted of 138 items in total and took around 20 min to respond to.

### Analyses

Initially, the quantitative data was analyzed, after which the written responses were analyzed qualitatively to deepen the understanding of the HCPs’ ratings and views of PCC. Quantitative data was analyzed with descriptive and analytical statistics and the qualitative data from the written responses to the four study-specific questions at the annual education day were analyzed using content analysis.^25^

#### Statistical analysis

Initially, we performed descriptive statistics to explore and describe the quantitative data. Five items were negatively worded, and all statistical calculations were conducted after reversing those items (items 7, 8, 9, 10, 12). Sum scores were calculated for each of the two P-CAT subscales, based on Sjögren et al.’s two scales,^23^ after comparing Cronbach’s alpha between those two subscales and subscales proposed by Edvardsson et al.^22^ and others proposed by Rökstad et al.^24^ For calculations of sum scores, missing data were replaced based on person-mean imputation,^26^ if the missing data did not exceed 20%.^27^ Analytical statistics were performed with linear regression analysis. Independent variables were dichotomized above/below median value for continuous variables (age, time in the profession, time at the unit). Sex and targeted PCC education were dichotomous variables and education was a categorical variable, as was profession. Three outcomes—P-CAT total sum score, P-CAT subscale I, and P-CAT subscale II—were used as dependent variables in separate linear regressions. Background factors were included as independent variables and both univariable and multivariable regressions were estimated. The decision to include independent variables in the multivariable model was based on the independent variable’s explanatory power in the univariable analyses, with a significance level set at *p* ⩾ .05. The variable PCC education was judged to be of theoretical importance and was included in the multivariable analysis regardless of the result in the univariable analysis. A *p*-value ⩽ .05 was considered statistically significant. Statistical analyses were performed using IBM SPSS Statistics, version 28 (IBM Corp., Armonk, NY, USA) and the environment R, version 3.6.1 (R Foundation, GNU General Public License).

#### Qualitative analysis

A qualitative content analysis was performed to analyze the study-specific questions with open-ended written responses about PCC.^25^ On average, the responses were 1 or 2 sentences long, ranging from a couple of words to several sentences. Moreover, the content varied; some were quite exhaustive stories, while others were more concise, content-rich responses. In total, there were 170 responses by 41 HCPs to the four questions (1-4 responses/HCP). Initially, all answers were read closely to obtain an overview. The answers were then read again to identify and code parts of relevance for the study aim. The analysis was manifest, with as little interpretation as possible. After the initial reading of the answers and sorting into codes for text with similar content, the text segments were read again to identify overarching themes for the text segments, and the initial codes were merged together under common themes. Two of the researchers were involved in the analysis process and the other researchers confirmed the themes.

## Results

### Participants

In total, 85 HCPs of the total sample of 231 replied to parts of the survey (37% response rate), but only 51 of the responders replied to the P-CAT questions, giving a 22% response rate (51/231) for this part of the survey. Most of the participants who responded to the P-CAT questions were women (80.4%), with ages ranging between 23 and 72 years old (mean age: 44). Almost half of the responders were registered nurses (47.1%) (Table 2). The non-responders’ characteristics (*n* = 34) are presented in Table 2.

**Table 2.** Characteristics of the participants—total and separated P-CAT responders/non-responders.

Characteristics	Statistics/Category	Total *n* = 85 n (SD/%)	Responders *n* = 51 *n* (SD/%)	Non-responders *n* = 34 *n* (SD/%)
Age	Mean (SD)	44 (11.72)	43.71 12.15)	44.58 (12.23)
	Median (IQR)	44 (17.00)	41 (18.00)	44.50 (16.75)
	Range	23–72	23-72	26-66
Sex	Man	11 (12.6%)	9 (17.6%)	2 (7.4%)
	Woman	75 (86.2%)	41 (80.4%)	25 (92.6%)
	Other	0 (0.0%)	0	0
	Do not want to state	1 (1.1%)	1 (2%)	0
	Missing	7	0	7
Educational attainment	Higher secondary	19 (21.6%)	11 (21.6%)	8 (28.6%)
	Higher bachelor	43 (48.9%)	24 (47.1%)	12 (42.9%)
	Higher specialist/master	19 (21.6%)	11 (21.6%)	6 (21.4%)
	Other	7 (8.0%)	5 (9.8%)	2 (7.1%)
	Missing	6	0	6
Profession	Registered nurse	40 (45.5%)	24 (47.1%)	13 (46.4%)
	Physician	12 (13.6%)	8 (15.7%)	2 (7.1%)
	Assistant Nurse	19 (21.6%)	13 (25.5%)	6 (21.4%)
	Other*	17 (19.2%)	6 (11.8%)	7 (25.0%)
	Missing	6	0	6
Time working within the profession (years)	Mean (SD)	14 (12.35)	14 (12.20)	14.34 (14.18)
	Median (IQR)	10 (17.50)	10.50 (16.75)	8 (20.00)
	Range	0.5-47	1-47	0.5-45
Time working at the unit (years)	Mean (SD)	9.93 (10.55)	8.60 (9.55)	11.67 (12.79)
	Median (IQR)	4.75 (14.00)	4 (10.25)	4.75 (18.00)
	Range	0.40, 42.00	0.4-40	0.5-42
Taken part in targeted PCC education	Yes	30 (38.0%)	19 (37.3%)	9 (47.4%)
	No	41 (51.9%)	27 (52.9%)	7 (36.8%)
	Don’t know	8 (10.1%)	5 (9.8%)	3 (15.8%)
	Missing	15	0	15

Column percentages presented; missing cases excluded from the analyses.

* Other professions: physiotherapists, dieticians, counsellors, managers or administrative HCPs.

### Healthcare Professionals’ ratings of person-centred care according to P-CAT

The mean score obtained were 45.31 (SD: 7.88, range 28-62) for the total scale of P-CAT, 28.41 (SD: 5.32, range 16-39) for subscale one and 16.90 (SD: 4.73, range 8-24) for subscale two. The items with the highest ratings, that is, items which HCPs agreed completely or agreed with were “assessment of patients’ needs is undertaken on a daily basis” (74.5% agreed completely plus agreed; mean: 4.0), “we often discuss how to give PCC” (76.4 % agreed completely plus agreed; mean: 3.96) and “we have formal team meetings to discuss patients’ care” (70.6% agreed completely plus agreed; mean: 3.84). Items the participants rated lower were as follows: 31.4% agreed that “I simply do not have the time to provide PCC”; 41.2% agreed completely or agreed that “the environment is chaotic” and 31.4 % agreed completely or agreed with that “we have to get the work done before we can worry about a homelike environment” and 27.5% responded agree completely or agree with that “this organization prevents me from providing PCC” (Table 3).

**Table 3.** Responses to all P-CAT items %(n), Mean, (SD) and range.

Item	Agree completely % (*n*)	Agree % (*n*)	Neither agree nor disagree % (*n*)	Disagree % (*n*)	Disagree completely % (*n*)	Missing (*n*)	Mean (SD) range
1. We often discuss how to give PCC	33.3 (17)	43.1 (22)	13.7 (7)	5.9 (3)	3.9 (2)	34	3.96 (1.04) 1-5
2. We have formal team meetings to discuss patients’ care	35.3 (18)	35.3 (18)	13.7 (7)	9.8 (5)	5.9 (3)	34	3.84 (1.19 1-5
3. The life history of the patients is formally used in the care plans we use	13.7 (7)	51.0 (26)	21.6 (11)	9.8 (5)	3.9 (2)	34	3.61 (0.98) 1-5
4. The quality of the interaction between HCPs and patients is more important than getting the tasks done	7.8 (4)	39.2 (20)	31.4 (16)	19.6 (10)	0 (0)	35	3.36 (0.90) 2-5
5. We are free to alter work routines based on patients’ preferences	11.8 (6)	37.3 (19)	37.3 (19)	11.8 (6)	2.0 (1)	34	3.45 (0.92) 1-5
6. Patients are offered the opportunity to be involved in individualized everyday activities	15.7 (8)	29.4 (15)	25.5 (13)	23.5 (12)	2.0 (1)	36	3.35 (1.09) 1-5
7. I simply do not have the time to provide PCC	0 (0)	31.4 (16)	21.6 (11)	21.6 (11)	23.5 (12)	35	3.38 (1.18) 2-5
8. The environment feels chaotic	2.0 (1)	39.2 (20)	3.9 (2)	31.4 (16)	23.5 (12)	34	3.35 (1.28) 1-5
9. We have to get the work done before we can worry about a homelike environment	5.9 (3)	25.5 (13)	27.5 (14)	27.5 (14)	11.8 (6)	35	3.14 (1.13) 1-5
10. This organisation prevents me from providing PCC	2.0 (1)	25.5 (13)	17.6 (9)	21.6 (11)	31.4 (16)	35	3.56 (1.25) 1-5
11. Assessment of patients’ needs is undertaken on a daily basis	39.2 (20)	35.3 (18)	15.7 (8)	5.9 (3)	3.9 (2)	34	4.00 (1.08) 1-5
12. It is hard for patients in this facility to find their way around	2.0 (1)	13.7 (7)	11.8 (6)	21.6 (11)	43.1 (22)	38	3.98 (1.19) 1-5
13. Patients are able to access outside space as they wish	35.3 (18)	15.7 (8)	5.9 (3)	13.7 (7)	19.6 (10)	39	3.37 (1.64) 1-5

Column percentages presented; missing cases excluded from the analyses.

### Health care professionals’ ratings of PCC and associations with individual characteristics

For P-CAT subscale I, age was a significant explanatory factor in the univariable analyses, and an age of over 44 years old gave an average of 4.3 higher score than those 44 years old and younger (B = 4.30; CI:1.22-7.38; *p* = .00). This result was in line with those of the multivariable model, in which a significant association was seen between a higher age and higher PCC scores (B: 4.44; CI: 1.53–7.35; *p* = .00) and between having received PCC education within the research project and higher ratings of PCC (B: 3.45; CI: 0.46–6.64; *p* = .02) (Table 4). For P-CAT subscale II, no significant associations were seen.

**Table 4.** Results from regression univariable and multivariable analyses for P-CAT subscale 1.

	Univariable			Multivariable		
Term	Beta	95%-CI	*P*-value^a^	Beta	95%-CI	*P*-value^a^
Sex: Female (ref: Male)	.40	(–3.61, 4.41)	.842			
Age: >44 years (ref: ⩽44 years)	4.30	(1.22, 7.38)	**.007**	4.44	(1.53, 7.35)	**.004**
Time in profession: >10 years (ref: ⩽10 years)	1.28	(–1.72, 4.28)	.396			
Time at the unit: ⩾10 years (ref:<10 years)	–.20	(–3.23, 2.84)	.898			
PCC-education : Yes (ref: No)	1.94	(–1.13, 5.02)	.210	3.45	(0.46, 6.64)	**.025**
Education: Higher education (ref: secondary higher)	–1.79	(–5.66, 2.07)	.356			
Education: Higher education specialist/master level (ref: higher secondary)	–.18	(–4.71, 4.35)	.936			
Education: Other (ref: higher secondary)	3.00	(–2.73, 8.73)	.297			
Profession: MD (ref: R.N.)	1.42	(–2.95, 5.78)	.517			
Profession: Nurse assistant (ref: R.N.)	2.84	(–0.84, 6.52)	.127			
Profession: Other (ref: R.N.)	1.25	(–5.29, 7.79)	.702			

a Significance if *p* ⩽ .05.Bold numbers in the table are ⩽.05.

Workplace was included in the analyses, but not presented.

### Healthcare professionals’ views of PCC

HCPs’ views about PCC in the context of allo-HCT were expressed through the open-ended answers and the following three themes were identified: *Patient’s narrative and capability, Patient involvement in care* and *Room for improvement of PCC.*

#### Patient’s narrative and capability

HCPs described PCC as the patient being seen as an individual and a person who is capable, with their own resources.

PCC for me is to see the person behind the illness — a person with their own resources.

The participants described listening as a foundation for PCC and for allowing the possibility to actually “see” and get to know the patient. They also stated how listening and seeing the patient as a capable individual—as knowledgeable and in charge of their own care— created a feeling of safety and increased the patient’s confidence, thereby facilitating opportunities to take more responsibility and own their illness.

The person feels listened to and seen as a human being — and safe.

#### Patient involvement in care

HCPs described how the patient should be involved in their own care by receiving information and being aware of what is happening, to be able to influence their care. Furthermore, participants stated that PCC involves a partnership between the patient and healthcare and described how working together with the patient improves the care. The HCPs expressed how the patient’s story and communication with the patient are essential factors contributing to the achievement of a partnership and patient involvement in the care. They further voiced how involvement and communication together represent a prerequisite for a trusting relationship between the patient and healthcare, which is the basis for a partnership in care.

PCC for me is to base the work on the patient’s needs and what the patient wishes for. Good communication is the foundation of a good relationship.

The participants reported that PCC increases patient and family member involvement in care, and they had experienced how that resulted in higher care satisfaction and enabled more tailored care according to patients’ unique individual needs. HCPs mentioned that when the patient is more informed and involved in their care, the patient’s resources were utilized more, which resulted in patients being more well-equipped for self-care. The participants also saw a benefit regarding PCC for themselves in that a shift in focus away from tasks needing to be done leads to more creative and interesting work.

The patient becomes more involved in the care and it is easier when the patient and the staff work toward the same goal. It also makes it easier when the staff get a better understanding of the patient, which leads to a better relationship and a more confident patient.

### Room for improvement of PCC

The participants did think that they currently work in accordance with PCC, but also expressed that there is room for improvement. They reported that the level of person-centredness depends on who is working at the time and that the involvement of patients in their own care could be better. They also voiced an awareness of having to involve the patient in the care and acknowledge the patient’s own resources—and mentioned that they do try but there is a lack of continuity and frequent changes of staff. The staff turnover was described in terms of the incorporation of new co-workers, as well as a lack of continuity in the patient care, where changes of staff for the individual patient were sometimes implemented to ease the workload for staff, without considering what is best for the patient. The HCPs also reported the lack of time available to work in a person-centred way.

We work person-centred to a certain extent, but it can definitely be developed, we need to involve the patient more. There is no time to work person-centred, but person-centredness can also create more time if it is used from the start.

## Discussion

This study investigated HCPs’ ratings and views of PCC in allo-HCT care, as well as associations with individual characteristics and targeted PCC education. The results of the current study show that the level of PCC in the context of allo-HCT is high, but they indicate that there is room for improvement. In the written responses to the study-specific open-ended questions, HCPs described PCC as seeing the patient as a capable individual and involving the patient in care — and they also highlighted the room for improvement of PCC.

HCPs had high ratings of person-centredness in care in this study and in the written responses the HCPs also stated that they partly work in accordance with PCC. The mean total P-CAT score (45.31) in this study is slightly lower than in another study focusing on HCPs in 12 nursing homes, in which the mean total P-CAT score was 50.3.^28^ However, in another study in 175 Norwegian nursing homes the mean total P-CAT score was in line with the one obtained in the current study.^29^ The HCPs’ views that they work in accordance with PCC in this study are in line with a study at two university hospitals in Denmark which showed that HCPs in general perceived a PCC culture, although 25% of the HCPs reported that patients were not included in assessing, planning and evaluating the care.^30^ In the present study, HCPs had lower ratings regarding the organizational and environmental aspects which enable provision of PCC—reflecting (i) how they do not have time to provide PCC, (ii) the chaotic nature of the care environment, and (iii) how the organization prevents them from providing PCC. These results are in line with a review study^31^ about conceptualizations of “person-centredness” for serious illness, based mainly on HCPs’ and patients’ perceptions. The review highlights how important it is for a care organization to enable care continuity to achieve PCC by valuing, involving and supporting social networks of patients—supporting patients in continuation of normality, preserving their self-identity and promoting quality of life.^31^

The results further show that a higher age of the participants was associated with higher ratings of PCC, as was targeted PCC education. The higher ratings of level of PCC associated with a higher age of the participant could be explained by the fact that HCPs with higher age have more experience in the profession and more extensive life experience in general, resulting in them rating their level of PCC as higher. This possible explanation would be in line with Benner’s Novice to Expert theory which presents an understanding of how a nurse new in the profession develops skills and understanding of a practice over time.^32^ However, there was no significant association between years in the profession and level of PCC. Another plausible explanation is that younger HCPs have more knowledge and greater awareness of the meaning of PCC given that it has been included more and more in education curriculums over time. The state of PCC education in higher education in Sweden is ongoing albeit with a variable degree to which it is included in curricula in different programmes across education providers, as well as an uncertainty around the meaning of PCC.^11,12^ Internationally, there is a growing recognition of the need for PCC in care and the need to educate HCPs about PCC, promoted through for example, national policies and frameworks. There is however no overview of the international state of how PCC is integrated into curricula and practice. A national standard for inclusion of PCC in education for HCPs could bring about more equal knowledge of PCC, a shared view of PCC and enable implementation in practice.

In the present study, the targeted education about PCC was associated with HCPs’ higher ratings of PCC, which is positive since HCPs rate their level of PCC as higher and consider that they work in accordance with PCC even after they have undergone a targeted education about PCC providing a greater understanding and general consensus regarding PCC. Previous research has shown that professional training and education is essential for successful implementation of PCC.^33^ However, even though HCPs undergo a targeted education and rate their level of PCC as high, this cannot be considered as a guarantee of high levels of PCC actually occurring in the delivery of care. Healthcare provided in accordance with PCC does not just happen—the work culture needs to be changed to include HCPs’ prerequisites, such as their values, beliefs and competence, as well as skills to provide PCC.^34^ Additionally, a care environment that includes the physical environment and organizational systems that are supportive of PCC is essential for a culture change to accommodate PCC.^34^

In the written responses to the study-specific open-ended questions, HCPs described PCC as the patient being seen as a capable individual with their own resources and mentioned that PCC increases patient and family member involvement, resulting in higher care satisfaction and tailored care that meets patients’ individual needs. HCPs described views of PCC that contained the foundations of PCC—patients’ narratives, capability and involvement, as well as the partnership between HCPs and patients. However, in the written responses, the aspect of PCC in which the partnership is safeguarded by shared decision-making and documentation in a health plan is less described.^35^ It may be that HCPs can theoretically explain what PCC is, especially following an annual education day focusing on PCC, but this is not equivalent to it being incorporated into the clinical work and actually adhered to in care. A common language of person-centredness is emerging, but there is a need for more explicit recommendations on how PCC can be operationalized in everyday practice situations.^34^ Giusti et al.^31^ stress that HCPs need support to adapt skills, communication, routines and environments for individual patients. Furthermore, PCC demands a shared philosophy of care, satisfactory leadership, support from colleagues and continuing education. The authors suggest that appointing a care coordinator to each patient or arranging for nurses to provide additional guidance following a physician visit may be practical steps for achieving PCC.^31^

HCPs stated in the written responses that they partly work according to PCC, but that there is room for improvement. The results from the survey indicate that the organizational support and care environment for working in accordance with PCC can be improved. The HCPs’ stated both in the survey and in their written responses to the open-ended questions that they do not have time to provide PCC. These results are in line with previous studies in which the organizational prerequisites and time to provide PCC have been highlighted.^30,33,36^ In the results from the survey, HCPs had higher ratings for often discussing patients’ care, discussing how to give PCC and assessment of patients’ needs, which may all be carried out in a person-centred manner, but may primarily be carried out with a medical focus. In one study, nurses working with allo-HCT reported that due to a highly medical-technical environment and medical focus in allo-HCT care, nursing became fragmented and opportunities for PCC—such as care planning or preparing patients for discharge and management of self-care—were limited.^37^ The HCPs’ views of PCC in this study are in line with a previous study about HCPs’ and patients’ views and experiences of PCC in oncology, in which additionally caring, compassion and empathy were reported as also being important parts of PCC.^38^ In addition, patients in another study stressed the importance of compassion, a positive nurse-patient relationship and clinically competent care to promote patients’ empowerment, participation in care and positive attitude toward recovery.^39^ Furthermore, it has been shown that patients’ experiences of caring and person-centredness are associated with perceived quality of nursing care, with HCPs’ knowledge and communication, as well as environmental support, being significantly associated with patients’ perceptions of nursing care quality.^40^

## Limitations

The response rate for this study was rather low (22%), although it should be noted that this figure is not remarkably low compared to other survey studies targeting HCPs^30^ It may be that the non-responders do not share the ratings and views of PCC presented in this study. A plausible reason for not responding to the survey reflects the ever-present dilemma in healthcare nowadays—lack of time. Another reason may be that the survey also contained questions about organizational readiness, including work situation and job satisfaction; hence, it was quite an extensive survey. HCPs may have considered that the survey content did not concern them. Furthermore, no power calculation for the sample size was done for this study, which could be considered a limitation regarding the possibility of generalization of the study results. A limitation of the data is that the answers to the open-ended study-specific questions were from HCPs at only one of the two participating centres, which may be of significance for the results. Furthermore, the written answers to the open-ended questions were gathered in connection with an annual educational day for staff focusing on PCC, and the open-ended study-specific questions were answered after they had received lectures and had discussions about PCC. This may of course have influenced their answers, but it may also have given them a better understanding of the pillars of PCC before responding about the state of PCC at their clinic. Despite these shortcomings, the study provides valuable knowledge, transferable to similar care settings, about healthcare professionals’ ratings and views of person-centred care in allo-HCT, and associations with individual characteristics and targeted PCC education, and potential room for improvement is highlighted.

In previous studies, P-CAT has been divided into different subscales.^22-24^ Therefore, Cronbach’s alpha was calculated for the different previously used subscales, and the subscales with the best Cronbach’s alpha for P-CAT (0.77 for subscale 1 and 0.79 for subscale 2) based on Sjögren et al.’s subscale^23^ were selected for the current study. Self-reporting data can be affected by an external bias caused by social desirability, which can be minimized by ensuring anonymity and confidentiality.^41^ It may be that the results of this study are overly positive given social desirability and the wish to work in accordance with PCC. Furthermore, allo-HCT involves very specific treatment and care. Additionally, the study sites are specialized in providing care for haematological diseases. However, the results of this study are considered transferable to other contexts where allo-HCTs are performed. Despite methodological limitations, this study provides novel and important knowledge about HCPs’ ratings and views of PCC in allo-HCT. It also reveals associations with individual characteristics and targeted PCC education.

## Conclusion

HCPs’ rated level of PCC in allo-HCT is high regarding discussing and assessing patients’ needs, but there is room for improvement of organizational and environmental aspects. The ratings of PCC were higher amongst HCPs that had taken part in the targeted PCC education. HCPs describe views of PCC partly including the foundations of PCC—patient’s narrative, capability and involvement. They also state that they partly work according to PCC, but that there is room for improvement. The organization could enable the provision of PCC with regard to HCPs time to provide PCC and by focusing on the care environment to make it more homelike and less chaotic. A clearer and shared view of the meaning of PCC—for example, achieved by targeted education on how to work in accordance with PCC—may increase HCPs’ ratings of PCC in allo-HCT.
